# Electromyography: Processing, Muscles' Electric Signal Analysis, and Use in Myofunctional Orthodontics

**DOI:** 10.7759/cureus.50773

**Published:** 2023-12-19

**Authors:** Shweta Tagore, Amit Reche, Priyanka Paul, Mihika Deshpande

**Affiliations:** 1 Public Health Dentistry, Sharad Pawar Dental College and Hospital, Datta Meghe Institute of Higher Education and Research, Wardha, IND

**Keywords:** myofunctional therapy, electrical signal, orthodontic therapy, electrical activity, electromyography, surface electromyography

## Abstract

Electromyography, commonly known as EMG, utilizes superficial or needle electrodes to record and analyze the fundamental electrical characteristics of skeletal muscles, determining whether the muscles are contracting. The motor unit, which consists of a collection of group muscle fibers and the motor neurons that govern them, is the structural basis of EMG. Three types of electrode are used in EMG which are needle electrode, fine wire electrode, and surface electrode. A significant amount of literature indicates that the correction of muscle function affects the relationships between teeth within the same jaw and between the jaws on opposing sides. The mechanism of action in myofunctional appliance therapy is linked to neuromuscular and skeletal adaptations resulting from altered function in the orofacial region. Both myofunctional therapy and orthodontics aim to address abnormal muscular behavior, restore abnormal muscle activity, and maintain proper alignment in various areas, including the lips, lower jaw, and tongue. This knowledge is essential for functions such as swallowing, speaking, chewing, and respiration as well as for minimizing incorrect movements and positioning. This article aims to describe the application of surface EMG as a diagnosis tool for assessing muscle activities in various orthodontic disorders, such as class II malocclusion open bite, crossbite, maxillary constriction, cleft lip and palate (CLP), and temporomandibular dysfunction, in patients. The electrodes used in EMG can be utilized to detect bioelectric activity in the muscles of the jaws and abnormalities in jaw movement. Analyzing EMG data is vital for obtaining a comprehensive understanding of the masticatory muscle system.

## Introduction and background

Electromyography, often known as EMG, employs superficial or needle electrodes to record and analyze the basic electrical characteristics of skeletal muscle to ascertain whether or not the muscle is contracting. It is frequently used in both clinical and research contexts. It is also crucial for the identification of facial muscle during neuromuscular orthodontic therapy and for the management of face discomfort brought on by the use of myofunctional appliances [1].

Myofunctional orofacial anomalies are conditions in which there are abnormalities in the development, structure, or function of the orofacial muscles. Such problems can result in temporomandibular joint (TMJ) dysfunction, jaw malocclusions, and other abnormalities in the orofacial area. Malocclusions may be brought on by myofunctional abnormalities, which include breathing, deglutination, and biting pattern disorders. Examples of clinically prevalent conditions that can be brought on by genetic and/or environmental causes include infantile mouth breathing and infantile swallowing [2-4]. Functional therapy and orthodontics aim to address unusual muscular behavior [5] as follows: restoring muscle function; maintaining proper function of oral and maxillofacial regions, including the lips, lower jaw, and tongue; knowledge in deglutination, speaking, mastication, and respiration; and minimizing movement that is incorrect and/or position. The most suitable period to start treatment is during the pre-puberty stages with high sutural growth. The placement of teeth and the appearance of the dental arch can indeed be influenced by various factors, among which the peripheral oral musculature and labial posture are notable contributors [6]. EMG is a valuable tool for monitoring muscular activity during functional treatment, playing a crucial role in directing therapy. It is a diagnostic technique that records the electrical activity of muscles. The initial attempt to integrate EMG into dentistry was undertaken by Robert E. Moyers [7]. Other methods, including plaster models, lateral cephalometry, and cone beam computed tomography (CBCT), are conventional techniques that do not provide immediate results regarding muscle contraction. EMG, on the other hand, generates electrical signals from masticatory muscles and can detect abnormal muscle function. It has been recognized that the balance of muscles in the same jaw and the opposing jaw significantly affects the optimal connections between teeth. In the context of orthodontics, the important mandible elevator muscles include the masseter and temporalis muscles as well as the lateral and medial pterygoid muscles. Additionally, the genioglossus muscle significantly impacts facial form [7].

This narrative review article aims to provide information about EMG processing and analysis, the evaluation of masticatory muscle activity in various myofunctional disorders, and myofunctional therapy (MFT). It also briefly includes limitations and factors affecting EMG in patients.

## Review

Physiology of EMG

**Figure 1:**
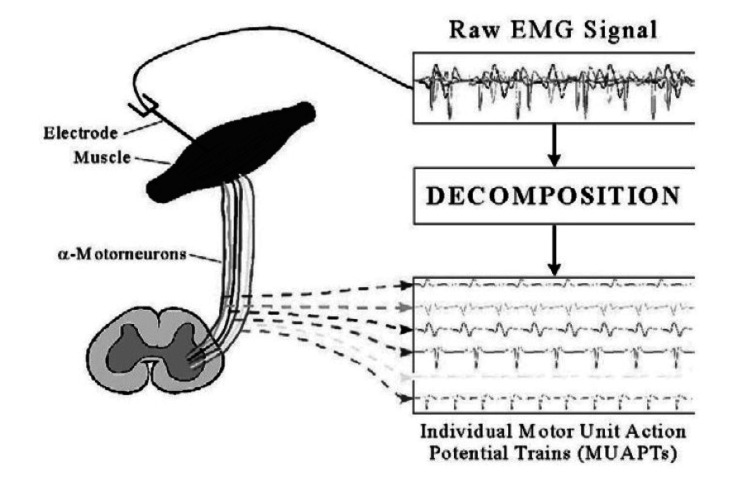
EMG signal and decomposition of MUAPs EMG: electromyography; MUAPs: motor unit action potentials Image Credit: Raez et al. [1]

The fundamental structure of EMG is the motor unit, which is composed of a group of muscle fibers and the motor neurons that control them. When a motor neuron starts an action potential, the muscle fibers of the whole motor unit contract. This electric potential decreases in nearby tissue [8]. The total extracellular potential of a motor unit's muscle fiber action potentials is known as the motor unit action potential (MUAP). The quality of the EMG signal is affected by two main problems during detection and recording. The signal-to-noise ratio, which measures the energy difference between the EMG signals and noise signals, is an initial problem. Noise is a common term for electrical impulses that are not necessary for the EMG signal [1]. Both invasive and non-invasive electrodes have been used to record muscle signals. When EMG is recorded using electrodes directly linked to the skin, the EMG signal is a composite of all the muscle fiber action potentials developing in the muscles beneath the skin. The EMG signal may occasionally display both positive and negative voltage due to the irregular spacing between these action potentials. The MUAP is measured using either a skin surface electrode applied externally (non-invasive) or a needle electrode injected intramuscularly (invasive). It is the sum of all the muscle fiber action potentials from a single motor unit [1]. The signal is captured and amplified by the electrode. There may be further stages of amplification beyond the initial stage, which is often a differential amplifier signal that can be generated across the plasma membrane before being shown or saved. There may be differences between the fluids found within and outside of the cell. A muscle fiber depolarizes and contracts in response to stimulation from the neuron as a result of the signal moving along its surface. Ion mobility and an electric field are also produced by this depolarization surrounding the entire muscle fiber. An EMG signal is made up of a train of MUAP, which depicts the reaction of the muscle to brain stimulation [1]. The MUAP serves as the filter for the EMG signal, which is often disoriented in appearance. The neuron pulses are referred to as the "impulse process" in this context and are typically conceived of as a Poisson process [9]. Figure 1 shows the procedure for obtaining an EMG signal as well as the decomposition to produce MUAPs.

Types of electrodes

The electrodes of an EMG come in two major varieties: electrodes on the surface and implanted electrodes. Thin wire electrodes and needle electrodes are two other types of implanted electrodes. The following explains the three electrodes (surface, needle, and fine wire).

The Electrode of the Needle

These are the methods used the most commonly in neuromuscular evaluations. The needle electrode's exposed tip is used as a detecting surface. In the tube of the cannula, there is an insulated wire. In terms of signal quality, needle electrodes are superior to other varieties that are now available.

The Electrode of Fine Wire

Wire electrodes can be manufactured from any small-diameter, strong, insulated, and extremely non-oxidizing wire. Common materials include alloy and noble metals silver, platinum, nickel, and chromium. Extremely thin and simple to implant and remove from muscles in the skeleton are fine wire electrodes [10].

Surface Electrodes

For the non-intrusive monitoring and detection of EMG information, surface EMG (sEMG) electrodes are used. Electrolytic conduction, as per the theory, results in the formation of a chemical balance between the body's skin and the detecting surface when the current is directed into the electrode [11].

EMG electrode placement

sEMG electrodes should be positioned along the muscle's lengthwise midline, between the tendinous insertion and the motor unit [12]. The electrodes' or detecting surfaces' centers must be spaced apart by 1-2 cm. The length of the muscle fibers should be aligned to the longitudinal plane of the electrodes.

EMG recording procedure in patients

**Figure 2:**
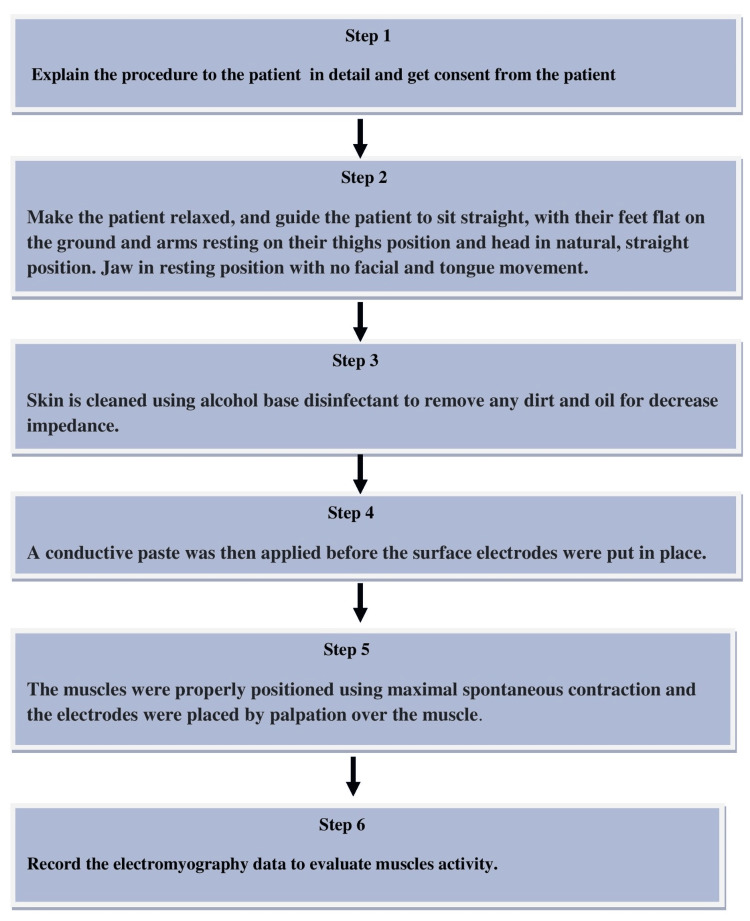
Steps of EMG procedure recording in a patient EMG: electromyography Image Credit: Shweta Tagore

The location of the recording should be in an insulated room with minimum light to prevent outside electrical interference. The protocol for the EMG recording procedure in patients is described in a stepwise manner in Figure 2.

EMG is used for the assessment of muscle activity in MFT

Myofunctional appliances, employed in interceptive orthodontic treatments, are most effective when used in growing children, particularly those who have not yet undergone the pubertal growth spurt, as this developmental phase offers a crucial phase for influencing facial and dental structures. The goal is to address malocclusions and other orthodontic issues early on, taking into consideration the ongoing growth to guide the proper alignment of teeth and jaws [13]. Functional appliances are used to correct poor mastication and improper tongue and jaw posture and treat TMJ disorder. Many studies have demonstrated that functional appliances, both permanent and removable, produce intraoral forces that alter the masticatory muscles' postural and functional activity [14,15].

For optimum craniofacial growth and development, the person must be able to breathe through their nose while keeping their lips closed and their tongue in a normal position over their palate. Incompetent lips can press and move the upper incisors [16]. Several fundamental muscles, including the masseter, anterior and posterior temporalis, anterior digastric, and sternocleidomastoid, are involved in chewing, swallowing, and maintaining head posture. These muscles are essential for many aspects of oral and cervical function [17]. The temporalis frequently forms the basis for mandibular balance and postural control, whereas the masseter is mostly used during grinding and chewing [18]. The muscles that sEMG often assesses are the masseter and anterior temporalis. The electrical activity of the masticatory muscles can be observed both passively (at rest, during maximum or submaximal voluntary clenching) and actively (during opening and closing of the oral cavity, anterior, posterior, and lateral movement of the jaw, eating, deglutition, or talking) [19].

EMG has been utilized widely by orthodontists in the diagnosis and treatment of muscle functional hyperactivity and hypoactivity, abnormal occlusion positions, muscle imbalance, spasms, fatigue, weakness of muscles of mastication, and muscle posture activity [20-22].

EMG Activity Evaluated in Various Abnormal Occlusion Positions

Class II skeletal malocclusions frequently involve mandibular retrognathism, characterized by the backward positioning of the lower jaw. This condition is commonly associated with anterior displacement of the maxilla, an elevated posterior maxillary vertical dimension, a posteriorly situated mandibular fossa, and maxillary constriction [23]. A study on the investigation of class II malocclusion and muscle activity during maximal biting in the intercuspal position (ICP) shows that individuals with class II malocclusion exhibited lower EMG activity in the masseter and temporal muscles compared to those with normal occlusion. Notably, class II children showed a more pronounced reduction in EMG activity during chewing than normal occlusion, especially in the masseter muscle. The findings revealed strong positive correlations between EMG activity during maximal biting and chewing for both muscles in both occlusion groups [24].

Mandibular prognathism concerning the maxilla and/or cranial base characterizes class III malocclusion, a dentofacial deformity associated with growth [25]. Comparing class III malocclusion to class I malocclusion, there is a decrease in the EMG activity of the temporal and masseter muscles, which is caused by reduced muscle contraction [26].

In the primary and early mixed dentition, posterior crossbite is a frequent malocclusion that affects 8-22% of orthodontic patients [27] and 5-15% of the general population [28]. In 71-84% of people with a posterior crossbite, there is a unilateral posterior crossbite (UPCB) accompanied by a functional displacement of the jaw [29]. The objective of maxillary growth is to extend the cross widths of the maxilla by opening the mid-palatal suture, which is one of the treatment options for the repair of skeletal restriction of the upper jaw. In 2018, Michelotti et al. conducted a study to evaluate jaw muscle function in children with UPCB pre and post rapid maxillary expansion (RME) using sEMG. They examined 29 children with UPCB and 40 controls. The study found that UPCB did not lead to asymmetric muscle activation during functional tasks and RME treatment did not improve muscle symmetry [30].

A deep bite is described as a malocclusion where the vertical overlap of the front teeth exceeds the ideal value, often associated with diminished vertical facial dimensions, and is commonly diagnosed when there is an overbite exceeding 3 mm [31]. In addition, a study showed that for both the deep bite patients and the control group, the kinematic variables about the chewing pattern which were related to masticatory efficiency and recorded with both hard and soft boluses were dependent on the bolus hardness [32]. Thus, there is no difference between deep bite patients and normal people in terms of their ability to apply load. Put another way, even though deep bite patients' muscle activation during chewing was noticeably higher than that of the control group, they were nevertheless able to adjust their chewing pattern to the bolus consistency. Additionally, as previous studies have shown, the examination of the chewing cycles reveals that the malocclusion considerably restricts the lateral component of the closure stroke, resulting in more vertical and repeating patterns [33]. According to this study and earlier research using functional removable appliances, functional therapy combined with a functional-producing bite can significantly minimize the muscle hyperactivity of individuals with deep bites when they are chewing [34].

The orthodontic strategy for addressing deep bite cases should differ from that employed for dental crowding, given the impact of malocclusion on neuromuscular control. Emphasizing the potential side effects is crucial, as a recurrence of muscular hyperactivity may lead to a relapse of malocclusion. Patient awareness of this risk enables timely identification and treatment, preventing potential serious damage to teeth and other structures in the stomatognathic system, which can be irreversible in older individuals [35].

An anterior open bite is described as the absence of vertical overlap between the upper and lower front teeth when the posterior teeth are in contact. Tongue thrusting is considered a common cause of open bite. The tongue is a robust muscular organ that plays a crucial role in directing the eruption of teeth, shaping the bite, and preserving the form and stability of the dental arch through its force on the teeth [36]. The habit of tongue thrusting exerts pressure on the upper and lower dental arch intermittently, which can lead to an open bite. A study on the effects of changes in occlusion vertical dimension (OVD) on perioral musculature during tongue thrusting showed that, as the OVD increased, there was a statistically significant rise in the EMG activity of both the upper and lower lips. This study also observed an increase in swallow duration and EMG activity as OVD increased, suggesting that more effort was required for both swallowing and achieving lip sealing [37]. The treatment of open bite typically requires a multidisciplinary approach, combining MFT to address tongue posture and orthodontic treatment for correcting malocclusion. Oral myofunctional therapy (OMT) involves neuromuscular re-education of the muscles, aiming to correct the rest posture and movements of the tongue, lips, and cheeks, as well as patterns of breathing, mastication, and swallowing. This comprehensive therapy includes speech therapy and physiotherapy for the tongue and lips to improve swallowing patterns. Additionally, breaking habits are addressed through the use of orthodontic appliances. The primary objective of OMT is to heighten awareness of incorrect tongue positioning and functioning, with the ultimate goal of harmonizing orofacial functions through the learning of a new, more physiological swallowing pattern [38]. The study was conducted on patients with skeletal anterior open bite (SAOB), with EMG activity evaluated before and after a three-month OMT. This includes the activity of the anterior temporalis, masseter muscle, and anterior digastric. Additionally, EMG activity associated with the closed mouth state, including the activity of the upper orbicularis and mentalis muscles, was recorded. Before treatment, EMG activity at rest showed a significant increase in the anterior temporalis and mentalis muscles in SAOB subjects compared to normal subjects. However, the activity of the anterior temporalis and masseter muscle decreased in the ICP. Furthermore, the activity of the upper orbicularis and mentalis muscle significantly increased during lip sealing and swallowing, indicating that the oral myofunctional status (OMS) features of SAOB mainly include aberrant swallowing patterns and weak masticatory muscles. After the three-month OMT, the EMG activity of masticatory muscles significantly increased, while that of perioral muscles decreased. This suggests that OMT might contribute to the normalization of OMS by strengthening the muscles associated with mastication in the ICP and relaxing the perioral muscles during lip sealing [39].

In cleft lip and palate (CLP), aberrant mandible and maxilla growth can lead to severe malalignment, impairing the maxillofacial musculature's function [40]. From infancy through adulthood, interdisciplinary surgical and nonsurgical care is necessary for the successful rehabilitation of cleft patients [41]. In several published investigations, sEMG has been employed to measure the electric potentials of the masticatory muscles and the superior orbicularis oris in individuals with a CLP-related abnormalities [42]. In individuals with lip and palate defects, sEMG was also employed to monitor surgical or orthodontic treatments and detect muscle electrical potentials, as investigated by Sabbag et al. [43]. The electrical signals of the masseter and temporalis muscles were analyzed in 32 nonsyndromic complete unilateral CLP patients when their palates were surgically repaired using one of two distinct palate repair procedures. The electric potentials of the masticatory muscles were recorded both at rest and during eating. The researchers discovered that identical masseter and temporalis EMG activity was observed during functional and at rest after both one-stage and two-stage palate closure. The results may be justified by discrepancies in the assessment methodologies and the electromyographic evaluation tests, even though they were assessed properly. Standardized procedures in electromyographic assessment are essential, according to Ferrario et al. [44], to reduce potential biases and inaccuracies, in both the collecting and processing of the EMG signal. 

EMG Assessment of Muscle Hyperactivity and Hypoactivity in Myofunctional Disorder

Muscle activity can be changed by different malocclusion, and this might serve as a risk factor for more severe malocclusion. Patients with varying malocclusions may exhibit varying levels of masticatory muscle efficacy. Certain malocclusion patients may adopt an incorrect masticatory position that could hinder orthodontic therapy. The dimensions of the male and female craniofacial structures differ. It has been established that masticatory muscle activity and craniofacial morphology are related [45]. The masseter and temporalis muscles are important masticatory muscles that work by elevating, pollinating, and retracting the mandible. Therefore, malaligned teeth may have an impact on the force used during chewing and clenching. The masseter is primarily responsible for elevating the mandible, while the temporalis assists in elevation and retraction [8].

Studies have revealed that the masseter and temporalis muscles of male and female groups are activated differently during basal/rest, post basal, chewing, and clenching. Significantly higher levels of right temporalis muscle activity were observed in the male group and distinct malocclusion groups. There were no discernible gender or malocclusion group differences in the right masseter muscle activity. In comparison to the female group, the male group's left masseter muscle displayed more chewing activity. The EMG activity of the patient in the class I malocclusion group was higher than that of the class II and class III malocclusion groups [46]. Ferrario et al. observed that, during normal and resting jaw closure, the temporalis muscle exhibited lower activity in younger patients with class II division I malocclusion. In response to this reduced activity, the posterior region of the temporalis muscle showed increased activation, suggesting a compensatory response. According to the study, over time, this diminished function of the temporalis muscle may contribute to the development of a more severe malocclusion [47].

Muscle Spasm, Fatigue, and Weakness Assessment in Temporomandibular Disorders (TMD) Using EMG

TMD refers to a series of clinical signs of impairment of the temporomandibular system. This system comprises the TMJ, masticatory muscles, and tendons and ligaments surrounding the TMJ [48]. A person's posture, lifestyle, functional variables (including posture and neural activity), psychological factors (like stress), and/or any combination of these elements can be the cause of TMD [49]. The standard technique used to evaluate a patient with TMD in terms of both their physical and psychosocial features is the Research Diagnostic Criteria for Temporomandibular Disorder. The symptoms of TMD include altered TMJ function and discomfort [50]. The three most common symptoms, referred to as the "TMD triad," are limited range of motion, joint crepitus, and pain in the mandibular joint and/or masticatory muscles [51].

Sherman employed EMG to find activity in the masseteric regions of individuals whose main complaint was TMJ pain. The patients were divided into four groups according to whether they had a history of clenching or bruxism as well as if they showed physical signs of evident TMJ dysfunction. The findings revealed that while the group with mixed problems significantly differed from the normal group, 16 patients with evident TMJ disorders, who had no history of clenching or bruxism, showed no significant quantitative or clinical differences from the normal group. Therefore, the presence of TMJ issues alone does not increase the degree of muscle contraction; rather, noticeable muscle contraction occurs when TMJ is combined with clenching and bruxism [52]. Pain in the TMJ's temporal muscle is always taken into consideration when a spasm is detected. When there are occlusion interferences, spontaneous activation of the muscles is seen during rest. Myofunctional appliances were discovered to change the patterns of muscle activation in pain-free, healthy individuals by Lobbezoo et al. According to the study, splints increased EMG activity in the masseter muscle but decreased it in the anterior temporalis muscle [53]. Research conducted by Ferrario et al. revealed that there was no significant difference in the average potential of men and women. But while clenching, males showed higher electromyographic levels and more contact during centric occlusion. Hamada et al. conducted EMG tests on the masseter and anterior temporalis muscle to ascertain the best course of treatment for bruxism patients who report fatigue, soreness, and muscular pain. The findings showed that the increased activity had worn out the masticatory muscles [54].

Biofeedback reduces muscle discomfort and regulates muscle tension by providing recipients with extrinsic feedback they would not normally receive, enabling them to acquire supplementary information in addition to the intrinsic feedback they receive [55]. Elevated EMG levels in the jaw muscles are associated with stress and tension. Tsai et al. found a significant increase in the EMG activity of masticatory muscles under induced stress conditions. Consequently, progressive muscle relaxation is considered an effective therapeutic approach for TMD to enhance the range of motion and alleviate pain [56].

Masticatory Muscle Imbalance Evaluated in EMG

The relationship between bilateral muscular activity and muscle balance signifies muscle equilibrium. TMD is a common symptom of masticatory muscle imbalance, which is typically seen in patients with skeletal transversal issues [57]. Muscle balance study suggests that occlusal stability is more important than skeletal morphology [58]. However, skeletal and dental posterior crossbites have been documented to affect muscle balance [59]. In addition, patients with skeletal malocclusion show indifferent symmetry of the anterior temporal and less symmetry of masseter muscle activity [60]. The indifferent anterior temporal activity might contribute to the patient's neuromuscular adaptation from their mild skeletal asymmetry. However, TMD has been associated with posterior crossbite, anterior open bite, Angle class II and III malocclusions, and increased overjet [61]. Some acute malocclusions (sudden occlusal changes) might also arise as a consequence of a joint or muscle disorder. Even though a recent study has concluded that orthodontic treatment cannot prevent TMD to a great degree, the results showed that the difference in TMD incidence between orthodontically treated and untreated subjects was not statistically significant. This suggests that TMD signs and symptoms may not be directly related to malocclusion and its treatment [62].

Correlation of Muscle Activity in Head and Tongue Posture 

The interaction between tongue positioning, head posture, and swallowing is intricate, as each variable significantly influences the others [63]. In this interdependent relationship, proper tongue placement is crucial for maintaining balance in oral and facial muscles, while head posture impacts the alignment of these structures [64]. The coordination of these factors is particularly vital during the complex motor activity of swallowing, where any discrepancies in tongue positioning or head posture may contribute to difficulties in the swallowing process.

Swallowing is more comfortable when upright, but the difficulty increases with head or neck flexion or extension, leading to a reduced opening in the airway or esophagus [65]. In terms of anatomy, the tongue is closely connected to the hyoid bone, forming relationships with both the suprahyoid and infrahyoid muscles [66]. In the initial stage of swallowing and phonation, these muscles work together to coordinate movements in the jaw and tongue [67]. EMG revealed electrical activity in the omohyoid muscle and the anterior belly of the digastric muscle during various tongue movements. These muscles play a crucial role in establishing a proper connection between the tongue and the head (neck) during flexion, extension, and rotation of the neck and cervical region [68]. Suprahyoid muscles are vital for head posture and balance. Suprahyoid muscles are vital for head posture and balance. It's associated with issues like tongue thrusting, open bite, shallow palate, and jaw underdevelopment. Furthermore, it may contribute to temporomandibular pain in cases of TMD. Correcting tongue posture often involves a combination of neurophysiotherapy and oral myofunctional rehabilitation therapy. A global postural rehabilitation program, initially developed by a French physical therapist in the 1980s and now applied globally, is also employed for corrective purposes [69]. Its methods and exercises center around motor control, activating antagonist muscles, and integrating sensory input to enhance mobility, flexibility, muscle strength, and overall function. This aims to achieve muscle balance and postural symmetry.

Fixed mechanotherapy in malocclusions

In adult patients with dentoalveolar malocclusion, fixed mechanotherapy is the preferred choice for orthodontic treatment. Fixed mechanotherapy involves the use of pre-adjusted edgewise appliances, and tooth movement is achieved using archwires and elastics. Functional appliances, utilized in the correction of malocclusion, alter the neuromuscular environment of the dentition and associated bones [70]. The resulting skeletal alterations are attributed to morphologic adaptations in response to altered muscular tone and a change in the direction of traction exerted by the masticatory muscles [71]. Fixed functional appliances are worn for a long period, involve continuous displacement, and are therefore expected to elicit a greater and more rapid neuromuscular response. The study on class II division I malocclusion assessed neuromuscular adaptation with a rigid fixed functional appliance. It prompted definite responses in the anterior temporalis and masseter muscles. After six months, there was sufficient adaptation, with a significant decrease in muscle activity during saliva swallowing and clenching. The study supports the role of viscoelastic muscle elements and increased lip strength in bone remodeling with these appliances. The proposal suggests that using a fixed functional dental appliance triggers motor reprogramming and postural changes, leading to a growth response and impacting jaw and facial development in orthodontic treatments [72].

Factors affecting EMG activity in orofacial muscles

Electromyographic signals can be influenced physiologically by age, sex, temperature, stress, and skin thickness (electrical signal conduction is lower in thick skin or high subcutaneous fat) [73]. Saifuddin et al. [74] conducted a study comparing muscle activity during mastication in two recording sessions to resting muscle activity measured throughout the day and night, revealing that the masseter and temporalis muscles were least active during nighttime.

The accuracy and reliability of sEMG are affected by factors such as the electrode's location relative to the muscle under examination, skin resistance, and ambient noise [75]. To enhance skin-electrode contact and reduce skin resistance, it is advisable to remove hair, scrub the skin, and clean it with alcohol.

Limitations of EMG

While various muscles are involved in functions like chewing, swallowing, and phonation, EMG studies have predominantly concentrated on transient and bulk muscles due to their accessibility, larger size, and ease of location and the reliability of results they provide [75].

The masseter and anterior temporalis muscles are the most frequently assessed in sEMG because the electrodes used in this method lack high selectivity and can only detect signals from muscles that are near the skin's surface. This approach has limitations, such as the necessity of hair removal for recording the activity of the medial and posterior fibers of the temporalis muscles, which patients often find unacceptable, and the need to address anatomical challenges related to the pterygoid muscles. The primary goal of sEMG is to capture signals originating from a large number of muscle fibers in proximity to the detecting electrode. As a result, the analysis of these recordings is primarily focused on assessing general muscle activity, and the involvement of other specific muscles is challenging due to the limited selectivity of the electrodes.

## Conclusions

EMG plays a crucial role as a diagnostic tool in orthodontics and maxillofacial orthopedics. It is primarily employed to assess muscle function, monitor treatment progress, and comprehend the impact of orthodontic interventions on the stomatognathic system. This method records masticatory muscle activity in both resting and functional positions, contributing to the evaluation of MFT by detecting abnormal muscle activity and aiding in the management of myofunctional disorders.
